# Recent Advances in Metal-Organic Framework-Based Electrochemical Biosensing Applications

**DOI:** 10.3389/fbioe.2021.797067

**Published:** 2021-12-16

**Authors:** Mengjie Li, Guangyao Zhang, Andrews Boakye, Huining Chai, Lijun Qu, Xueji Zhang

**Affiliations:** ^1^ State Key Laboratory of Bio-Fibers and Eco-Textiles, Intelligent Wearable Engineering Research Center of Qingdao, Research Center for Intelligent and Wearable Technology, College of Textiles and Clothing, Qingdao University, Qingdao, China; ^2^ School of Environmental and Municipal Engineering, Qingdao University of Technology, Qingdao, China; ^3^ Institute of Materia Medica, Shandong First Medical University and Shandong Academy of Medical Sciences, Jinan, China; ^4^ School of Biomedical Engineering, Health Science Center, Shenzhen University, Shenzhen, China

**Keywords:** metal-organic framework, electrochemical biosensing, small biomolecules, biomacromolecules, pathogenic cells

## Abstract

In the face of complex environments, considerable effort has been made to accomplish sensitive, accurate and highly-effective detection of target analytes. Given the versatility of metal clusters and ligands, high porosity and large specific surface area, metal–organic framework (MOF) provides researchers with prospective solutions for the construction of biosensing platforms. Combined with the benefits of electrochemistry method such as fast response, low cost and simple operation, the untapped applications of MOF for biosensors are worthy to be exploited. Therefore, this review briefly summarizes the preparation methods of electroactive MOF, including synthesize with electroactive ligands/metal ions, functionalization of MOF with biomolecules and modification for MOF composites. Moreover, recent biosensing applications are highlighted in terms of small biomolecules, biomacromolecules, and pathogenic cells. We conclude with a discussion of future challenges and prospects in the field. It aims to offer researchers inspiration to address the issues appropriately in further investigations.

## Introduction

Rapid industrial developments and modern lifestyle have affected human health, for which people pay more attention to the dynamics of their physical condition (Pettinari et al., 2021). The observation of biological activities, the acquisition of biological information, and the discovery of life systems have fueled the development of biotechnologies, particularly biosensors (Zhao et al., 2021). Biological signals that are difficult to perceive are converted to readable data using optics (Sun et al., 2021a), photothermal (Zhou et al., 2020a), electrochemistry (Zhang et al., 2021b), electrochemiluminescence (Zhang et al., 2020), and other technologies. Electrochemical biosensors have thrived due to their high sensitivity, rapid response, and simplicity of operation (Selvam et al., 2021). To improve the applicability and sensibility of the biosensing system, the consumables and volumes of samples required for detection should be reduced as far as possible (Ma et al., 2021).

Metal–organic framework (MOF) is a hybrid material in which metal ions are bridged with organic ligands. A wide variety of central nodes and ligands has resulted in a vast library of coordination options. The adaptable network structure of MOF is well suited for functionalized modification, and the properties of MOF can be adjusted by tuning their topology and porosity (Pashazadeh-Panahi et al., 2021). The potential applications of MOF include gas storage and separation (Fan et al., 2021), heterogeneous catalysis (Wu and Zhao, 2017), sensing (Osman et al., 2019; Li et al., 2021c), drug delivery (Wang et al., 2020b), proton conductivity (Ye et al., 2020), etc. Although the chemical stability, conductivity, and catalytic site utilization of MOF have somewhat limited its electroactivity, the tunable nature and high mass-transfer efficiency make it ideal for electrochemical biosensing platforms (Wang et al., 2018). According to research, four modular MOFs with different stacking approaches exhibit specific heterogeneous electron transfer rates for ascorbic acid (AA), dopamine (DA), uric acid (UA), and serotonin (5-HT), which are related to metal nodes and heteroatomic cross-linkers (Ko et al., 2020). Shieh et al. (Shieh et al., 2015) first proposed a *de novo* method for embedding catalase in zeolitic imidazolate framework-90 (ZIF-90) crystals, which demonstrated that decreasing the pore size of MOF weakens the interaction between the matrix and enzymes and prevents the leaching of the contents. Integrating bioentities into MOF enhances the specificity of the biosensor, and the chemical mutability of MOF allows the molecular-level control of the sensing performance (Velásquez-Hernández et al., 2021). The ongoing outbreak of coronavirus disease 2019 (COVID-19) has become a global public health emergency (Wang et al., 2020a). As of November 2021, the number of reported infections worldwide has exceeded 252 million (World Health Organization, 2021), causing severe harm to human health and the global economy. MOF-based electrochemical sensors have been proposed for detecting viruses such as severe acute respiratory syndrome coronavirus 2 (SARS-CoV-2) (El-Sherif et al., 2021), human immunodeficiency virus (HIV) (Lu et al., 2021), hepatitis-C virus (HCV) (Sheta et al., 2020), which are essential for diagnostic treatment and delaying the spread of diseases.

**FIGURE 1:**
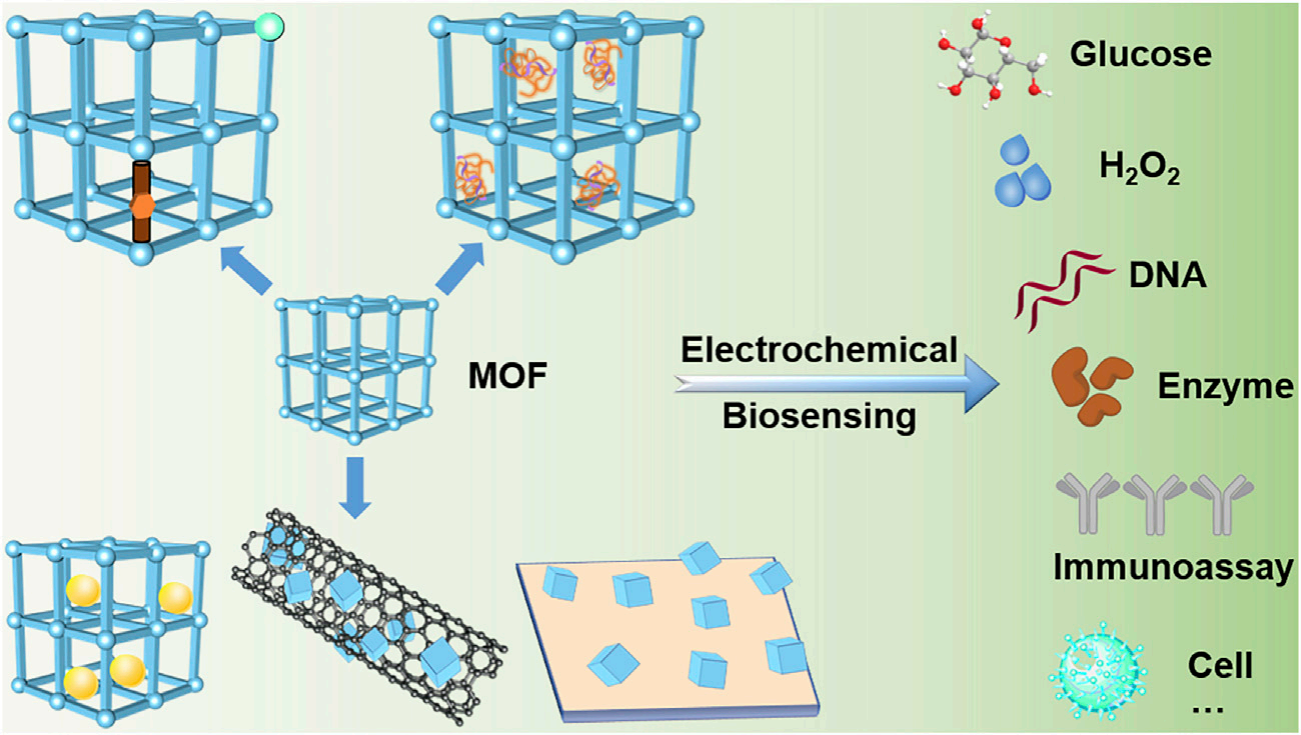
Illustration of the electroactive MOF designing strategies, including select redox ligands or metal centers, combine with biomolecules, and assembly of composites. The synthesized functional materials are used to detect small biomolecules, biomacromolecules, pathogenic bacteria/cells through electrochemical methods.

This review focuses on the application of electroactive MOF to electrochemical biosensing. First, three common strategies for preparing electroactive MOF are generalized: synthesizing using redox ligands or metal ions, combining with active guest molecules, and compounding with nanoparticles (NPs), carbon materials, polymers, and other materials (Figure 1). Second, examples of the functional MOF-materials used in electrochemical biosensors are summarized, including small molecule metabolites, neurotransmitters, nucleic acids, vitamins, antigens, enzymes, and pathogenic bacteria or cells. Subsequently, the trends and challenges in this field are discussed, concentrating on the structure–function relation in electroactive MOF, and on new designs for practical electrochemical sensing devices.

## Synthesis and Modification of MOFS for Electroactive Materials

Fully mobilizing the activity of MOF is usually a challenging task. The internal structure of MOF can be custom-designed by adjusting its topology, crystallinity, pore size, porosity, and other properties. Due to the ordered connections and stable coordination of MOF, the performance of the adopted highly active organic ligands or metal ions is no longer limited to specific forms. In comparison to common inorganic porous materials, the properties of MOF can be naturally and easily modified to prevent damage to guest molecules caused by complicated procedures. The heterostructures formed using MOFs with NPs or nanocarbon-based materials further compensates for some of the inherent defects of MOFs (Ling et al., 2020).

### Synthesis Using Organic Ligands or Metal Ions

Porphyrin is a popular candidate for the MOF organic unit due to its fine structure, distinct optical and electrical properties, and potent chemical catalytic activity (Zhang et al., 2016b). The MOF coordination structure can effectively modulate the electronic properties of porphyrin for molecular recognition and ion sensing applications (Zhang et al., 2016a; Zhang et al., 2021a). The coordination structure of MOF can effectively modulate the electronic properties of porphyrin for molecular recognition and ion sensing applications (Chen et al., 2021). For example, Zn^2+^ and 5,10,15,20-tetrakis-(4-carboxyphenyl)porphyrin (TCPP) were combined into two-dimensional (2D) nanosheets, ZnTCPP MOF. The elliptical flakes provide numerous reaction sites and reduce the energy required for the transition of the luminophore to its excited state (Han et al., 2021). Moreover, tetrathiafulvalene (TTF) is a strong π-electron donor with a conjugated polysulfide structure. It has been linked to four lanthanide metals (Tb, Dy, Ho, and Er) in different bridging patterns to form three-dimensional (3D) MOF (Su et al., 2019). In another study, the center of TTF was replaced with Ni to form a novel functional ligand, nickel bis(dithiolene-dibenzoic acid). As the core of the reaction, NiS_4_ can rapidly oxidize glucose to yield a signal response (Zhou et al., 2020b). Hollow nanospheres (HNSs) are NPs with voids in a solid shell, having a high specific surface area, low density, and large internal space. Designing MOF with mixed metal ion centers can improve its water stability and electroactivity (El-Sheikh et al., 2021). Li et al. doped Fe in bimetallic MOF containing Ni and Co to prepare Fe@NiCo-MOF HNSs. The Fe dopant changes the electronic structure of Ni and Co and affords a superior catalytic activity to Fe@NiCo-MOF HNSs comparing with the monometallic MOF (Li et al., 2020a).

### Functionalization of MOF With Biomolecules

The integration of MOF with biomolecules has shown broad prospects in drug delivery, cancer treatment, and biosensing (Dutta et al., 2019). As a mild and inexpensive immobilization substrate, MOF considerably improves the stability, tolerance, and recoverability of the enzyme (Zhuang et al., 2017). The position of macromolecules within microcrystals can be controlled by adjusting the topology of MOF, contributing to the encapsulation or diffusion effect of enzymes (Liang et al., 2018). Alternatively, the conformation of the aspartate protease was changed by the guidance of the organic medium. Then, it shifted into the pores of an Al-based MOF (MIL-101(Al)–NH_2_) with only half of its original volume, completing an effective binding without the loss of activity (Navarro-Sanchez et al., 2019). The performance of biomodified MOF is non-negligibly affected by external conditions such as temperature, light, acidity, and alkalinity. The interaction between Au and thiolated molecules allows DNA probes to grow on the Au electrode surface, forming DNA self-assembled monolayers (SAMs) that are sensitive but show low stability as a sensing element. To compensate for the stability deficiency, zeolitic imidazolate framework-8 (ZIF-8) was grown on an electrode surface modified with DNA SAMs, forming a favorable barrier outside the nanoprobe (Ma et al., 2019). Such MOF–biomolecule integration strategies reduce the activity loss of sensing materials during assembly, storage, transportation, and inspection operations, which have advanced their dissemination.

### Modification for MOF Composites

Diverse MOF-based composites often outperform single materials and are broadly exploited in biosensing. Li et al. (Li et al., 2020b) made DA to self-polymerize on the surface of Au NPs and then compounded with Fe-MOF to form Au@PDA@Fe-MOF (PDA = polydopamine). PDA can adjust the size of Au NPs and promote electron transfer together with Fe active centers. This aptamer sensor with dual amplified signals considerably improves the sensitivity of carcinoembryonic antigen (CEA) detection. In addition to their improved conductivity, MOF composited with carbon materials yield unexpected effects on stability and structural controllability. For instance, the uniformly stacked layered structure of graphene oxide (GO) achieves a larger specific surface area, thus promoting coupling interactions between different metal ions (Xing et al., 2021). Multiwalled carbon nanotubes (MWNTs) and Cu-MOF were assembled layer by layer via alternate solidification to form an electrodeposited film on a glassy carbon electrode (GCE). The nitrated MWNTs enhances the catalytic activity of the Cu-MOF and amplifies the signal (Wu et al., 2019b). Adding carbon dots (CDs) to the MOF precursor solution reduces its crystallization zone during growth, increasing the likelihood of binding MOF to the aptamer. CDs embedding increases the electroactivity of MOF, thereby accelerating the response to the target (Gu et al., 2019). A lot of MOFs are crystalline, which majorly limits their modification, assembly, and application. Many researchers have attempted to overcome this barrier by combining MOFs and polymers. Thakur et al. (Thakur et al., 2021) grew polymer monomers in the pores of the synthesized MOFs and verified that conjugate polymerization does not degrade the structure of MOFs. The addition of poly (3,4-ethylenedioxythiophene) (PEDOT) remarkably improves the inherently poor conductivity of Fe-BTC (BTC = 1,3,5-benzenetricarboxylic acid) MOF.

## Emerging Biosensing Applications of Electroactive MOF Materials

Endowing MOF with high electroactivity, biocompatibility, and adaptability considerably expands their applications in electrochemical biosensing. Because of their permanent porosity and high homogeneity, MOF can accommodate many guest molecules and control the molecule release process. Moreover, the abundance of reaction sites enables the instantaneous occurrence of large-scale reactions. These excellent properties have been exploited in advanced biosensors. The most recent advancements in biosensors fabricated using electroactive MOF materials are summarized in this section from the standpoint of biomarker categories.

### Small-Biomolecule Sensing

**FIGURE 2:**
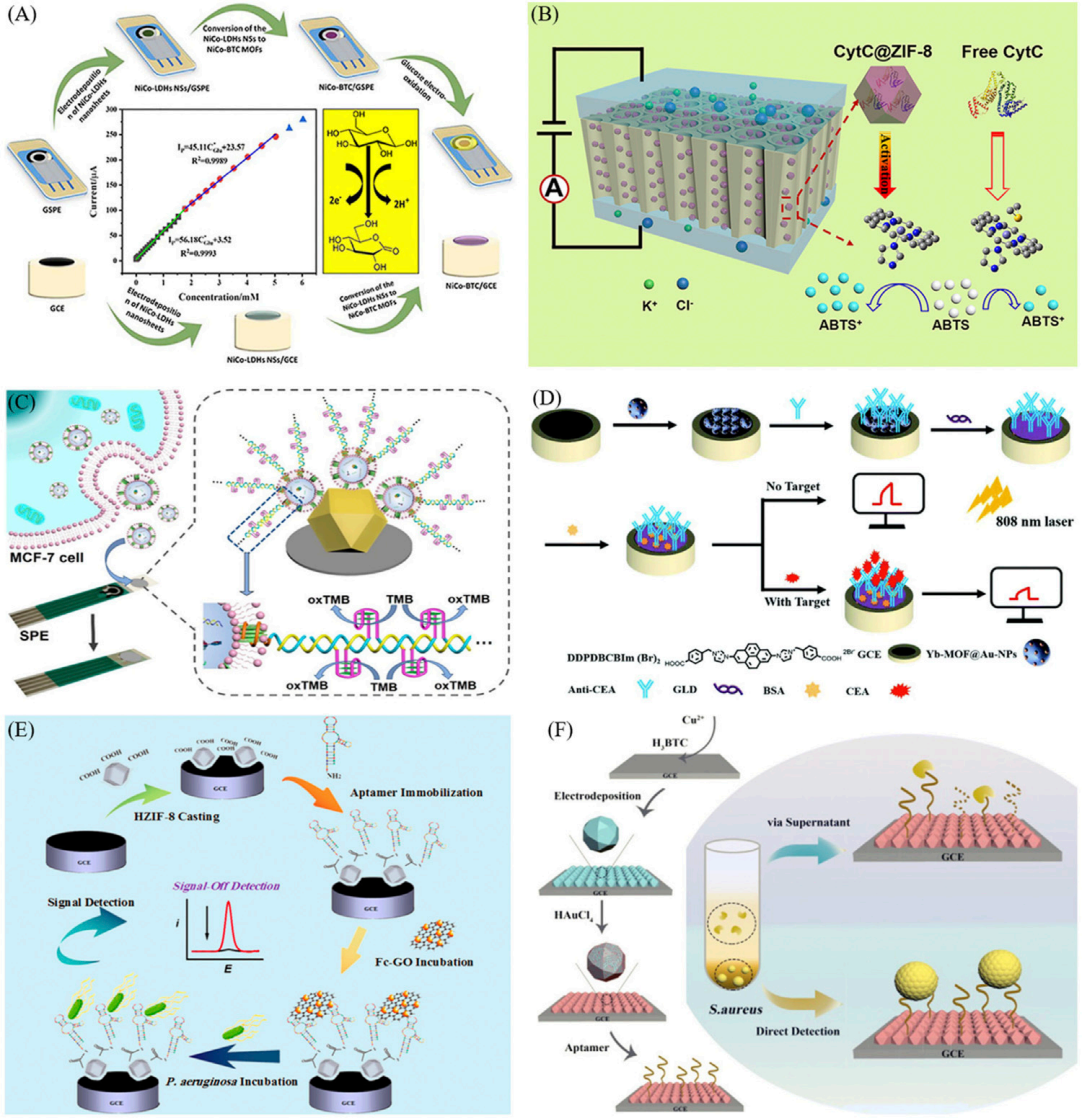
Biosensing applications of electroactive MOF materials in previous reports. **(A)** Rapid *in situ* synthesis of bimetallic MOF on the electrodes via a two-step method enables sensitive detection of glucose (Ezzati et al., 2020). **(B)** Stable and amplified peroxide-like enzyme activity is obtained by growing ZIF-8 encapsulating CytC in TiO_2_ nanochannels (Guo et al., 2020). **(C)** Modification of UiO-66 on fiber paper prompts the formation of DNAzyme as a signal amplifier for sensing exosomes (Liu et al., 2021). **(D)** Intense photocurrent of Yb-MOF@Au-NPs in near-infrared light for quantitative detection of CEA (Li et al., 2021a). **(E)** Modified ZIF-8 solid-loaded *P. aeruginosa* aptamer for detection of bacteria by signal off (Shahrokhian and Ranjbar, 2019). **(F)** Cu-MOF film enables dual detection of *S. aureus* and its characteristic secretions (Sun et al., 2021b).

Ni and Co. hydroxides are electrodeposited on carbon-based electrodes and subsequently coordinated with an acidic organic solution to obtain dispersed MOF flakes (Figure 2A). The synergistic interaction between the two metal centers intensifies the oxidation of glucose by the MOF (Ezzati et al., 2020). The advanced blood sensor analysis has been performed via 3D hollow fiber membranes (HFMs) (Wu et al., 2021). The subtle pore structure of HFMs can embed MOF mimics and biological enzymes with surprising blood-cell filtration ability. The polyaniline layer enhances the electrical signal outputs of multiple human health indicators (glucose, lactate, and cholesterol).

Ge’s group from Tsinghua University prepared ZIF-8 complexes wrapped with cytochrome C (CytC) in different sizes and morphologies (Feng et al., 2021). The relatively small size of CytC@ZIF-8 shows enhanced porosity and a large specific surface area, thereby reducing the contact resistance between the enzyme and substrate. Furthermore, CytC@ZIF-8 grown on the walls of TiO_2_ nanochannels form a successful MOF-in-nanochannel model for biosensing devices (Figure 2B). The oxidation process of CytC increases the electron loss from 2,2′-azino-bis(3-ethylbenzothiazoline-6-sulfonate), enabling the *in situ* monitoring of H_2_O_2_ concentration using CytC@ZIF-8/TiO_2_M (Guo et al., 2020). To mitigate the negative impact of the mechanical properties of the electrode on sensing, Shu et al. deposited carbon nanotubes (CNTs) on poly (dimethylsiloxane) (PDMS), providing a flexible substrate on which DA in C6 cells was monitored even during tensile or bending deformations (Shu et al., 2020). Recently, MOF was combined with a phase change material (PCM) for DA sensing. A microcapsule comprising paraffin, SiO_2_, and polypyrrole (PPy) was anchored with ZIF-8 from the core to the outer layer. The PCM autonomously regulates heat to control the temperature around the biosensor in real time, thus weakening the dependence of the sensing process on environmental conditions (Li et al., 2021b).

### Biomacromolecule Sensing

Exosomes are crucial for tumor diagnosis, migration, and growth, tissue damage repair, and neurodegenerative diseases. Gu et al. employed two MOF complexes (one as the anode and the other as the cathode) of a biofuel cell (Gu et al., 2021). Due to its large specific surface area, ZIF-8 enhances the catalytic activity of glucose dehydrogenase, acting as an outstanding electron producer. UiO-66 loaded with electroactive material (K_3_ [Fe(CN)_6_]) can be connected to exosomes, enhancing the electron connectivity and enabling it to accept numerous electrons. In such a self-powered sensor, the voltage is linearly and positively correlated with the exosome concentration. A fiber paper-based point-of-care (POC) sensor has been exploited based on the Zr-O-P interaction between exosomes and UiO-66 (Figure 2C). The specific recognition of the transmembrane protein CD63 and its aptamer triggers the DNA hybridization cascade reaction. The product can catalyze the oxidation of 3,3′,5,5′-tetramethylbenzidine (TMB) and decrease the response current (Liu et al., 2021).

Photoelectrochemistry (PEC) biosensors have attracted interest for high sensitivity and low background noise (Zhang et al., 2018). Li and co-workers synthesized an ionic liquid with a large conjugate system and coordinated it with Yb, forming Yb-MOF with a significant PEC response in the near-infrared region. Au NPs were modified on the Yb-MOF surface via *in situ* reduction, enhancing the absorption of incident light by Yb-MOF and accelerating the separation of electron–hole pairs (Li et al., 2021a). When the CEA antibody loaded on the surface of Yb-MOF@Au-NPs recognizes its target, the adsorbed CEA hinders the photoelectric conversion behavior and the photocurrent gradually fades (Figure 2D). Jiang et al. achieved a highly sensitive detection of the spike glycoprotein on the surface of SARS-CoV-2 using a photoactive material (Au NPs/Yb-TCPP). The plasma enhancement effect of Au NPs increases the photocurrent of Yb-TCPP by approximately 16 times. The PEC aptamer sensor based on Au NPs/Yb-TCPP demonstrates the high application potential of MOF materials in COVID-19 prevention (Jiang et al., 2021). Forming wrinkles on the surface of a Cu-based MOF (HKSUT-1) can obtain porous Cu_2_O-CuO flower. The specific binding between vascular endothelial growth factor 165 (VEGF165) and its aptamer will lead to a conformational change of DNA and introduce the Cu_2_O-CuO flower to the electrode. The unique photocurrent-polarity switching capability of Cu_2_O-CuO flower boosts the sensing efficiency of VEGF165 (Fu et al., 2020). These biosensing platforms have significantly promoted the use of MOF materials in clinical diagnosis and treatment (Wu et al., 2019a; Sheta et al., 2019).

### Pathogenic Cell Sensing

Bacterial contamination has become an urgent problem, especially in countries or regions with low economies and underdeveloped science and technology (Ghasemi et al., 2020). In one study, *Pseudomonas aeruginosa* (*P. aeruginosa*) levels in urine can be measured using a modified MOF (Figure 2E). The phenolic acid makes ZIF-8 tendency hydrophilic, promoting the attachment of *P. aeruginosa* aptamer. The specific recognition of *P. aeruginosa* separates the ferrocene-graphene oxide (Fc-GO) from the electrode, affording a gradually diminishing peak current (Shahrokhian and Ranjbar, 2019). Recently, a number of researches have shown that MOF-on-MOF yields functions that cannot be accomplished by a single MOF (Yao et al., 2019). The properties of MOF-on-MOF are influenced by the order of the cascade. For example, the crystallization of Tb-MOF-on-Fe-MOF is reduced by the limitation of Fe-MOF on the growth of Tb-MOF. Tb-MOF is more electroactive than Fe-MOF and readily interacts with the G-quadruplex. The precise quantification of carbohydrate antigen 125 (CA125) and living cancer cells using Tb-MOF-on-Fe-MOF shows the promising potential of MOF heterojunction materials in biosensing (Wang et al., 2019).

Xia et al. bound vancomycin with bovine serum albumin (BSA) on GCE to attract *Staphylococcus aureus* (*S. aureus*) through 1-(3-(dimethylamino)propyl)-3-ethylcarbodiimide hydrochloride/*N*-hydroxysuccinimide (EDC/NHS) chemistry. The 2D MOF solidified with *S. aureus* antibody was the second indication of accurate identification. The functional 2D MOF nanozyme exerts a potent catalytic effect on H_2_O_2_, generating hydroxyl radicals (·OH) that trigger the oxidation of *o*-phenylenediamine to complete the electrical signal transduction (Hu et al., 2021). Subsequently, a platform capable of the dual signal determination of *S. aureus* was established (Figure 2F). *In situ* reduction of Au NPs on the Cu-MOF surface enables it to load DNA aptamers. When aptamers encounter the micrococcal nuclease secreted by the pathogen, it lyses and detaches; consequently, the ion movement is accelerated and an upward signal is produced. In contrast, the specific binding between DNA aptamer and the pathogenic cells causes a decrease in the current (Sun et al., 2021b). High-performance detection systems for pathogenic substances represent a breakthrough in the application of MOFs in disease prevention, contributing to reduced medical costs.

## Conclusion and Perspective

In this review, we summarized the innovations and developments in the syntheses and applications of electroactive MOF materials. MOF has attracted much attention because of its flexible coordination structures, high modifiability, and favorable affinity for biomolecules. The catalytic activity is increased by the synergistic effect of the introduced multimetal sites and the design of novel organic ligands. The structure–activity relations among the pore size, topological configuration, morphology, and other features and properties of MOF have been gradually and intensively manipulated to achieve satisfactory functional outputs. In the curing of biomolecules, MOF can avoid the deactivation of biomolecules under external stimuli and its exposed active sites assist the diffusion of the substrate. The functional MOF composites compensate for the lack of conductivity and poor mechanical properties of single MOF. Researchers are working hard to fabricate low-cost, multi-functional, and environmentally friendly electroactive MOF materials.

The actual environment is often complex and variable, and the construction of biosensors faces many pressing problems. Future work should focus on the following aspects. 1) Designing new ligands or coordination of ligands, the properties of which better satisfy the actual needs, such as molecular structures that mimic the multibiological functions provided by nature (Alsharabasy et al., 2021). Regulating crystal growth while maintaining stability can yield (for example) hierarchical porous features that facilitate mass/electron transfer (Liu et al., 2020). 2) Although the high porosity endowment of MOF is desirable in redox reactions, the response time of the targets is sometimes long; alternatively, large volumes of samples are required. Both of these limitations reduce the sensitivity of biosensing. 3) When electroactive MOF functions as a signal emitter, the background response should be attenuated as much as possible so that an obvious signal can be collected even in extremely low concentrations of the analyte. 4) The miniaturization and integration of MOF sensing components promote the advancement of portable devices, and the market for wearable sensors and POC technology is in short supply (Yan et al., 2021a). Novel biosensors not only meet the daily healthcare needs of modern people but prevent disease transmission and save public medical resources by extending the biosensing applications of MOF to real-life (Yan et al., 2021b). Tailor-made MOFs have been used in drug delivery, cancer treatment, pollutant removal, supercapacitors, and other fields. We expect that MOF will further excel in electromagnetic wave absorption, air purification, rechargeable metal–air batteries, etc., providing solutions to dilemmas pertaining to global energy and the environment.

## References

[B1] Alsharabasy A. M. Pandit A. Farràs P. (2021). Recent Advances in the Design and Sensing Applications of Hemin/Coordination Polymer‐Based Nanocomposites. Adv. Mater. 33, 2003883. 10.1002/adma.202003883 33217074

[B2] Chen J. Zhu Y. Kaskel S. (2021). Porphyrin‐Based Metal-Organic Frameworks for Biomedical Applications. Angew. Chem. Int. Ed. 60, 5010–5035. 10.1002/anie.201909880 PMC7984248 31989749

[B3] Dutta S. Kim J. Hsieh P.-H. Hsu Y.-S. Kaneti Y. V. Shieh F.-K. (2019). Nanoarchitectonics of Biofunctionalized Metal–Organic Frameworks with Biological Macromolecules and Living Cells. Small Methods 311, 1900213. 10.1002/smtd.201900213

[B4] El-Sheikh S. M. Osman D. I. Ali O. I. Shousha W. G. Shoeib M. A. Shawky S. M. (2021). A Novel Ag/Zn Bimetallic MOF as a Superior Sensitive Biosensing Platform for HCV-RNA Electrochemical Detection. Appl. Surf. Sci. 562, 150202. 10.1016/j.apsusc.2021.150202

[B5] El-Sherif D. M. Abouzid M. Gaballah M. S. Ahmed A. A. Adeel M. Sheta S. M. (2021). New Approach in SARS-CoV-2 Surveillance Using Biosensor Technology: a Review. Environ. Sci. Pollut. Res. 10.1007/s11356-021-17096-z PMC8541810 34689274

[B6] Ezzati M. Shahrokhian S. Hosseini H. (2020). *In Situ* Two-Step Preparation of 3D NiCo-BTC MOFs on a Glassy Carbon Electrode and a Graphitic Screen Printed Electrode as Nonenzymatic Glucose-Sensing Platforms. ACS Sustain. Chem. Eng. 8, 14340–14352. 10.1021/acssuschemeng.0c03806

[B7] Fan W. Zhang X. Kang Z. Liu X. Sun D. (2021). Isoreticular Chemistry within Metal-Organic Frameworks for Gas Storage and Separation. Coord. Chem. Rev. 443, 213968. 10.1016/j.ccr.2021.213968

[B8] Feng Y. Zhao Y. Ge J. (2021). Impact of the Size Effect on Enzymatic Electrochemical Detection Based on Metal-Organic Frameworks. Analytica Chim. Acta 1149, 238191. 10.1016/j.aca.2020.12.066 33551062

[B9] Fu Y. Zou K. Liu M. Zhang X. Du C. Chen J. (2020). Highly Selective and Sensitive Photoelectrochemical Sensing Platform for VEGF165 Assay Based on the Switching of Photocurrent Polarity of CdS QDs by Porous Cu_2_O-CuO Flower. Anal. Chem. 92, 1189–1196. 10.1021/acs.analchem.9b04319 31769654

[B10] Ghasemi S. Bari M. R. Pirsa S. Amiri S. (2020). Use of Bacterial Cellulose Film Modified by polypyrrole/TiO_2_-Ag Nanocomposite for Detecting and Measuring the Growth of Pathogenic Bacteria. Carbohydr. Polym. 232, 115801. 10.1016/j.carbpol.2019.115801 31952600

[B11] Gu C. Guo C. Li Z. Wang M. Zhou N. He L. (2019). Bimetallic ZrHf-Based Metal-Organic Framework Embedded with Carbon Dots: Ultra-sensitive Platform for Early Diagnosis of HER2 and HER2-Overexpressed Living Cancer Cells. Biosens. Bioelectron. 134, 8–15. 10.1016/j.bios.2019.03.043 30952013

[B12] Gu C. Bai L. Pu L. Gai P. Li F. (2021). Highly Sensitive and Stable Self-Powered Biosensing for Exosomes Based on Dual Metal-Organic Frameworks Nanocarriers. Biosens. Bioelectron. 176, 112907. 10.1016/j.bios.2020.112907 33349536

[B13] Guo J. Yang L. Gao Z. Zhao C. Mei Y. Song Y.-Y. (2020). Insight of MOF Environment-dependent Enzyme Activity via MOFs-In-Nanochannels Configuration. ACS Catal. 10, 5949–5958. 10.1021/acscatal.0c00591

[B14] Han Q. Wang C. Liu P. Zhang G. Song L. Fu Y. (2021). Achieving Synergistically Enhanced Dual-Mode Electrochemiluminescent and Electrochemical Drug Sensors via a Multi-Effect Porphyrin-Based Metal-Organic Framework. Sensors Actuators B Chem. 330, 129388. 10.1016/j.snb.2020.129388

[B15] Hu W.-C. Pang J. Biswas S. Wang K. Wang C. Xia X.-H. (2021). Ultrasensitive Detection of Bacteria Using a 2D MOF Nanozyme-Amplified Electrochemical Detector. Anal. Chem. 93, 8544–8552. 10.1021/acs.analchem.1c01261 34097376

[B16] Jiang Z. W. Zhao T. T. Li C. M. Li Y. F. Huang C. Z. (2021). 2D MOF-Based Photoelectrochemical Aptasensor for SARS-CoV-2 Spike Glycoprotein Detection. ACS Appl. Mater. Inter. 13, 49754–49761. 10.1021/acsami.1c17574 34657424

[B17] Ko M. Mendecki L. Eagleton A. M. Durbin C. G. Stolz R. M. Meng Z. (2020). Employing Conductive Metal-Organic Frameworks for Voltammetric Detection of Neurochemicals. J. Am. Chem. Soc. 142, 11717–11733. 10.1021/jacs.9b13402 32155057

[B18] Li C. Li X.-J. Zhao Z.-Y. Li F.-L. Xue J.-Y. Niu Z. (2020a). Iron-doped NiCo-MOF Hollow Nanospheres for Enhanced Electrocatalytic Oxygen Evolution. Nanoscale 12, 14004–14010. 10.1039/d0nr01218a 32579652

[B19] Li J. Liu L. Ai Y. Liu Y. Sun H. Liang Q. (2020b). Self-Polymerized Dopamine-Decorated Au NPs and Coordinated with Fe-MOF as a Dual Binding Sites and Dual Signal-Amplifying Electrochemical Aptasensor for the Detection of CEA. ACS Appl. Mater. Inter. 12, 5500–5510. 10.1021/acsami.9b19161 31939286

[B20] Li H. Li Y. Zhang X. Liu P. He M. Li C. (2021a). Near-infrared Photoactive Yb-MOF Functionalized with a Large Conjugate Ionic Liquid: Synthesis and Application for Photoelectrochemical Immunosensing of Carcinoma Embryonic Antigen. Nanoscale 13, 9757–9765. 10.1039/d1nr01606g 34023865

[B21] Li J. Yu J. Sun Z. Liu H. Wang X. (2021b). Innovative Integration of Phase-Change Microcapsules with Metal-Organic Frameworks into an Intelligent Biosensing System for Enhancing Dopamine Detection. ACS Appl. Mater. Inter. 13, 41753–41772. 10.1021/acsami.1c13446 34459189

[B22] Li Y. Chai H. Lu Y. Tan W. Ma J. Zhang G. (2021c). Recent Progress and Applications of Optical/Electrochemical Sensors Based on Metal-Organic Frameworks for Water Environmental Detection. Chin. J. Anal. Chem. 49, 1619–1630. 10.19756/j.issn.0253-3820.210450

[B23] Liang W. Ricco R. Maddigan N. K. Dickinson R. P. Xu H. Li Q. (2018). Control of Structure Topology and Spatial Distribution of Biomacromolecules in Protein@ZIF-8 Biocomposites. Chem. Mater. 30, 1069–1077. 10.1021/acs.chemmater.7b04977

[B24] Ling P. Qian C. Yu J. Gao F. (2020). Artificial Nanozyme Based on Platinum Nanoparticles Anchored Metal-Organic Frameworks with Enhanced Electrocatalytic Activity for Detection of Telomeres Activity. Biosens. Bioelectron. 149, 111838. 10.1016/j.bios.2019.111838 31739109

[B25] Liu C.-S. Li J. Pang H. (2020). Metal-Organic Framework-Based Materials as an Emerging Platform for Advanced Electrochemical Sensing. Coord. Chem. Rev. 410, 213222. 10.1016/j.ccr.2020.213222

[B26] Liu X. Gao X. Yang L. Zhao Y. Li F. (2021). Metal-Organic Framework-Functionalized Paper-Based Electrochemical Biosensor for Ultrasensitive Exosome Assay. Anal. Chem. 93, 11792–11799. 10.1021/acs.analchem.1c02286 34407610

[B27] Lu Q. Su T. Shang Z. Jin D. Shu Y. Xu Q. (2021). Flexible Paper-Based Ni-MOF Composite/AuNPs/CNTs Film Electrode for HIV DNA Detection. Biosens. Bioelectron. 184, 113229. 10.1016/j.bios.2021.113229 33894427

[B28] Ma J. Chai W. Lu J. Tian T. Wu S. Yang Y. (2019). Coating a DNA Self-Assembled Monolayer with a Metal Organic Framework-Based Exoskeleton for Improved Sensing Performance. Analyst 144, 3539–3545. 10.1039/c9an00084d 31025665

[B29] Ma X. Zhang J. Zhang C. Yang X. Yu A. Huang Y. (2021). Targeting Enrichment and Correlation Studies of Glutathione and Homocysteine in IgAVN Patient Urine Based on a Core-Shell Zr-Based Metal-Organic Framework. ACS Appl. Mater. Inter. 13, 40070–40078. 10.1021/acsami.1c09967 34387999

[B30] Navarro J. Almora Barrios N. Lerma Berlanga B. Ruiz-Pernía J. J. Lorenz Fonfria V. A. Tuñón I. (2019). Translocation of Enzymes into a Mesoporous MOF for Enhanced Catalytic Activity under Extreme Conditions. Chem. Sci. 10, 4082–4088. 10.1039/c9sc00082h 31049190 PMC6469195

[B31] Osman D. I. El-Sheikh S. M. Sheta S. M. Ali O. I. Salem A. M. Shousha W. G. (2019). Nucleic Acids Biosensors Based on Metal-Organic Framework (MOF): Paving the Way to Clinical Laboratory Diagnosis. Biosens. Bioelectron. 141, 111451. 10.1016/j.bios.2019.111451 31252261

[B32] Pashazadeh-Panahi P. Belali S. Sohrabi H. Oroojalian F. Hashemzaei M. Mokhtarzadeh A. (2021). Metal-Organic Frameworks Conjugated with Biomolecules as Efficient Platforms for Development of Biosensors. Trac Trends Anal. Chem. 141, 116285. 10.1016/j.trac.2021.116285

[B33] Pettinari C. Pettinari R. Di Nicola C. Tombesi A. Scuri S. Marchetti F. (2021). Antimicrobial MOFs. Coord. Chem. Rev. 446, 214121. 10.1016/j.ccr.2021.214121

[B34] Selvam S. P. Kadam A. N. Maiyelvaganan K. R. Prakash M. Cho S. (2021). Novel SeS_2_-Loaded Co MOF with Au@PANI Comprised Electroanalytical Molecularly Imprinted Polymer-Based Disposable Sensor for Patulin Mycotoxin. Biosens. Bioelectron. 187, 113302. 10.1016/j.bios.2021.113302 34000454

[B35] Shahrokhian S. Ranjbar S. (2019). Development of a Sensitive Diagnostic Device Based on Zeolitic Imidazolate Frameworks-8 Using Ferrocene-Graphene Oxide as Electroactive Indicator for *Pseudomonas aeruginosa* Detection. ACS Sustain. Chem. Eng. 7, 12760–12769. 10.1021/acssuschemeng.9b01314

[B36] Sheta S. M. El-Sheikh S. M. Abd-Elzaher M. M. Ghanem M. L. Salem S. R. (2019). A Novel, Fast, High Sensitivity Biosensor for Supporting Therapeutic Decisions and Onset Actions for Chest Pain Cases. RSC Adv. 9, 20463–20471. 10.1039/C9RA03030A 35514688 PMC9065451

[B37] Sheta S. M. El-Sheikh S. M. Osman D. I. Salem A. M. Ali O. I. Harraz F. A. (2020). A Novel HCV Electrochemical Biosensor Based on a Polyaniline@Ni-MOF Nanocomposite. Dalton Trans. 49, 8918–8926. 10.1039/D0DT01408G 32555836

[B38] Shieh F.-K. Wang S.-C. Yen C.-I. Wu C.-C. Dutta S. Chou L.-Y. (2015). Imparting Functionality to Biocatalysts via Embedding Enzymes into Nanoporous Materials by a De Novo Approach: Size-Selective Sheltering of Catalase in Metal-Organic Framework Microcrystals. J. Am. Chem. Soc. 137, 4276–4279. 10.1021/ja513058h 25781479

[B39] Shu Y. Lu Q. Yuan F. Tao Q. Jin D. Yao H. (2020). Stretchable Electrochemical Biosensing Platform Based on Ni-MOF Composite/Au Nanoparticle-Coated Carbon Nanotubes for Real-Time Monitoring of Dopamine Released from Living Cells. ACS Appl. Mater. Inter. 12, 49480–49488. 10.1021/acsami.0c16060 33100007

[B40] Su J. Hu T.-H. Murase R. Wang H.-Y. D’Alessandro D. M. Kurmoo M. (2019). Redox Activities of Metal-Organic Frameworks Incorporating Rare-Earth Metal Chains and Tetrathiafulvalene Linkers. Inorg. Chem. 58, 3698–3706. 10.1021/acs.inorgchem.8b03299 30830770

[B41] Sun G. Xie Y. Sun L. Zhang H. (2021a). Lanthanide Upconversion and Downshifting Luminescence for Biomolecules Detection. Nanoscale Horiz. 6, 766–780. 10.1039/D1NH00299F 34569585

[B42] Sun Z. Peng Y. Wang M. Lin Y. Jalalah M. Alsareii S. A. (2021b). Electrochemical Deposition of Cu Metal-Organic Framework Films for the Dual Analysis of Pathogens. Anal. Chem. 93, 8994–9001. 10.1021/acs.analchem.1c01763 34151551

[B43] Thakur B. Karve V. V. Sun D. T. Semrau A. L. Weiß L. J. K. Grob L. (2021). An Investigation into the Intrinsic Peroxidase‐Like Activity of Fe‐MOFs and Fe‐MOFs/Polymer Composites. Adv. Mater. Technol. 6, 2001048. 10.1002/admt.202001048

[B44] Velásquez-Hernández M. d. J. Linares-Moreau M. Astria E. Carraro F. Alyami M. Z. Khashab N. M. (2021). Towards Applications of Bioentities@MOFs in Biomedicine. Coord. Chem. Rev. 429, 213651. 10.1016/j.ccr.2020.213651

[B45] Wang Z. Gui M. Asif M. Yu Y. Dong S. Wang H. (2018). A Facile Modular Approach to the 2D Oriented Assembly MOF Electrode for Non-enzymatic Sweat Biosensors. Nanoscale 10, 6629–6638. 10.1039/c8nr00798e 29578568

[B46] Wang M. Hu M. Li Z. He L. Song Y. Jia Q. (2019). Construction of Tb-MOF-On-Fe-MOF Conjugate as a Novel Platform for Ultrasensitive Detection of Carbohydrate Antigen 125 and Living Cancer Cells. Biosens. Bioelectron. 142, 111536. 10.1016/j.bios.2019.111536 31362204

[B47] Wang L. Wang Y. Ye D. Liu Q. (2020a). Review of the 2019 Novel Coronavirus (SARS-CoV-2) Based on Current Evidence. Int. J. Antimicrob. Agents 55, 105948. 10.1016/j.ijantimicag.2020.105948 32201353 PMC7156162

[B48] Wang Y. Yan J. Wen N. Xiong H. Cai S. He Q. (2020b). Metal-Organic Frameworks for Stimuli-Responsive Drug Delivery. Biomaterials 230, 119619. 10.1016/j.biomaterials.2019.119619 31757529

[B49] World Health Organization (2021). COVID-19 Weekly Epidemiological Update. Edition 66. Geneva: World Health Organization. Available at: https://covid19.who.int/ (Accessed November 20, 2021).

[B50] Wu C.-D. Zhao M. (2017). Incorporation of Molecular Catalysts in Metal-Organic Frameworks for Highly Efficient Heterogeneous Catalysis. Adv. Mater. 29, 1605446. 10.1002/adma.201605446 28256748

[B51] Wu H. Li M. Wang Z. Yu H. Han J. Xie G. (2019a). Highly Stable Ni-MOF Comprising Triphenylamine Moieties as a High-Performance Redox Indicator for Sensitive Aptasensor Construction. Analytica Chim. Acta 1049, 74–81. 10.1016/j.aca.2018.10.022 30612659

[B52] Wu L. Lu Z. Ye J. (2019b). Enzyme-free Glucose Sensor Based on Layer-By-Layer Electrodeposition of Multilayer Films of Multi-Walled Carbon Nanotubes and Cu-Based Metal Framework Modified Glassy Carbon Electrode. Biosens. Bioelectron. 135, 45–49. 10.1016/j.bios.2019.03.064 30991271

[B53] Wu H. Li T. Bao Y. Zhang X. Wang C. Wei C. (2021). MOF-enzyme Hybrid Nanosystem Decorated 3D Hollow Fiber Membranes for *In-Situ* Blood Separation and Biosensing Array. Biosens. Bioelectron. 190, 113413. 10.1016/j.bios.2021.113413 34116446

[B54] Xing Y. Zhang T. Lu N. Xu Z. Song Y. Liu Y. (2021). Catalytic Amplification Based on Hierarchical Heterogeneity Bimetal-Organic Nanostructures with Peroxidase-like Activity. Analytica Chim. Acta 1173, 338713. 10.1016/j.aca.2021.338713 34172151

[B55] Yan T. Zhang G. Chai H. Qu L. Zhang X. (2021a). Flexible Biosensors Based on Colorimetry, Fluorescence, and Electrochemistry for Point-of-Care Testing. Front. Bioeng. Biotechnol. 9, 753692. 10.3389/fbioe.2021.753692 34650963 PMC8505690

[B56] Yan T. Zhang G. Yu K. Li M. Qu L. Zhang X. (2021b). Smartphone-Based Point-of-Care Testing: Recent Progress and Applications. Prog. Chem. 10.7536/PC210407

[B57] Yao M. S. Xiu J. W. Huang Q. Q. Li W. H. Wu W. W. Wu A. Q. (2019). Van der Waals Heterostructured MOF‐on‐MOF Thin Films: Cascading Functionality to Realize Advanced Chemiresistive Sensing. Angew. Chem. Int. Ed. 58, 14915–14919. 10.1002/anie.201907772 31356720

[B58] Ye Y. Gong L. Xiang S. Zhang Z. Chen B. (2020). Metal-Organic Frameworks as a Versatile Platform for Proton Conductors. Adv. Mater. 32, 1907090. 10.1002/adma.201907090 32243018

[B59] Zhang G.-Y. Cai C. Cosnier S. Zeng H.-B. Zhang X.-J. Shan D. (2016a). Zirconium-Metalloporphyrin Frameworks as a Three-In-One Platform Possessing Oxygen Nanocage, Electron Media, and Bonding Site for Electrochemiluminescence Protein Kinase Activity Assay. Nanoscale 8, 11649–11657. 10.1039/c6nr01206j 27218308

[B60] Zhang G.-Y. Zhuang Y.-H. Shan D. Su G.-F. Cosnier S. Zhang X.-J. (2016b). Zirconium-Based Porphyrinic Metal-Organic Framework (PCN-222): Enhanced Photoelectrochemical Response and its Application for Label-free Phosphoprotein Detection. Anal. Chem. 88, 11207–11212. 10.1021/acs.analchem.6b03484 27750417

[B61] Zhang G. Shan D. Dong H. Cosnier S. Al-Ghanim K. A. Ahmad Z. (2018). DNA-Mediated Nanoscale Metal-Organic Frameworks for Ultrasensitive Photoelectrochemical Enzyme-free Immunoassay. Anal. Chem. 90, 12284–12291. 10.1021/acs.analchem.8b03762 30234968

[B62] Zhang G. Chai H. Tian M. Zhu S. Qu L. Zhang X. (2020). Zirconium-Metalloporphyrin Frameworks-Luminol Competitive Electrochemiluminescence for Ratiometric Detection of Polynucleotide Kinase Activity. Anal. Chem. 92, 7354–7362. 10.1021/acs.analchem.0c01262 32319281

[B63] Zhang G. Li M. Yu K. Chai H. Xu S. Xu T. (2021a). Two-Dimensional Metalloporphyrinic Framework Nanosheet-Based Dual-Mechanism-Driven Ratiometric Electrochemiluminescent Biosensing of Protein Kinase Activity. ACS Appl. Bio Mater. 4, 1616–1623. 10.1021/acsabm.0c01453 35014510

[B64] Zhang L. Qiao C. Cai X. Xia Z. Han J. Yang Q. (2021b). Microcalorimetry-Guided Pore-Microenvironment Optimization to Improve Sensitivity of Ni-MOF Electrochemical Biosensor for Chiral Galantamine. Chem. Eng. J. 426, 130730. 10.1016/j.cej.2021.130730

[B65] Zhao Y. Zeng H. Zhu X.-W. Lu W. Li D. (2021). Metal-Organic Frameworks as Photoluminescent Biosensing Platforms: Mechanisms and Applications. Chem. Soc. Rev. 50, 4484–4513. 10.1039/d0cs00955e 33595006

[B66] Zhou W. Hu K. Kwee S. Tang L. Wang Z. Xia J. (2020a). Gold Nanoparticle Aggregation-Induced Quantitative Photothermal Biosensing Using a Thermometer: A Simple and Universal Biosensing Platform. Anal. Chem. 92, 2739–2747. 10.1021/acs.analchem.9b04996 31977184

[B67] Zhou Y. Hu Q. Yu F. Ran G.-Y. Wang H.-Y. Shepherd N. D. (2020b). A Metal-Organic Framework Based on a Nickel Bis(dithiolene) Connector: Synthesis, Crystal Structure, and Application as an Electrochemical Glucose Sensor. J. Am. Chem. Soc. 142, 20313–20317. 10.1021/jacs.0c09009 33185447

[B68] Zhuang J. Young A. P. Tsung C. K. (2017). Integration of Biomolecules with Metal-Organic Frameworks. Small 13, 1700880. 10.1002/smll.201700880 28640560

